# Supplementary material for: Development of a Machine Learning Model to Distinguish between Ulcerative Colitis and Crohn’s Disease Using RNA Sequencing Data
Source: Diagnostics (Basel). 2021 Dec 15;11(12):2365. doi: 10.3390/diagnostics11122365 (PMC8700628; doi:10.3390/diagnostics11122365)
Supplement: Supplementary file 1 [file diagnostics-11-02365-s001.zip › diagnostics-1460658-supplementary/Supplmentary Table S1.pdf]

**Supplemental table S1.** Demographic information of human material

| | Patients with CD (n=94) | Patients with UC (n=33) |
|---|---|---|
| Male | 66 (77.6%) | 19 (22.4%) |
| Age, median years (range) | 30 (18-68) | 48 (18-69) |
| Disease duration, median year (range) | 4 (0-22) | 4 (0-24) |
| **Current drug use** | | |
| 5'-ASA | 55 (58.5%) | 21 (63.6%) |
| Glucocorticoids | 7 (7.4%) | 4 (12.1%) |
| Immunomodulator | 45 (47.9%) | 4 (12.1%) |
| Anti-TNF | 28 (29.8%) | 3 (9.1%) |
| Other biologics | 9 (9.6%) | 2 (6.1%) |
| **Smoking history** | | |
| Current smoker | 12 (12.8%) | 4 (12.5%) |
| Past smoker | 17 (18.1%) | 7 (21.9%) |
| Never | 65 (69.1%) | 21 (65.6%) |
| **Biopsy location** | | |
| **Normal** | | |
| Terminal ileum | 52 (80.0%) | 0 |
| Ascending colon | 8 (12.3%) | 0 |
| Transverse colon | 2 (3.1%) | 0 |

| | | |
|---|---|---|
| Descending colon | 2 (3.1%) | 0 |
| Sigmoid colon | 0 | 1 (7.7%) |
| Rectum | 1 (1.5%) | 12 (92.3%) |
| **Inflamed** | | |
| Terminal ileum | 10 (34.5%) | 0 |
| Ascending colon | 13 (44.8%) | 1 (5.0%) |
| Transverse colon | 0 | 0 |
| Descending colon | 2 (6.9%) | 0 |
| Sigmoid colon | 3 (10.3%) | 5 (25.0%) |
| Rectum | 1 (3.4%) | 14 (70.0%) |

CD = Crohn's disease, UC = ulcerative colitis, 5'-ASA= 5-aminosalicylic acid, TNF= tumour necrosis factor.
